# Utilizing whole genome sequencing to characterize central line-associated bloodstream infections due to *Staphylococcus epidermidis*

**DOI:** 10.1017/ice.2024.240

**Published:** 2025-03

**Authors:** Chunyi Zhou, Michael Wiley, Jessica Wiley, Kelly Cawcutt, Elizabeth Grashorn, Kathie Rogers, Emily McCutchen, Peter Iwen, Paul Fey, Mark Rupp

**Affiliations:** 1 Department of Pathology, Microbiology, and Immunology, University of Nebraska Medical Center, Omaha, NE, USA; 2 Nebraska Public Health Laboratory, Omaha, NE, USA; 3 PraesensBio, LLC, Omaha, NE, USA; 4 Division of Infectious Diseases, Department of Internal Medicine, University of Nebraska Medical Center, Omaha, NE, USA; 5 Department of Infection Control & Epidemiology, Nebraska Medicine, Omaha, NE, USA; 6 Clinical Microbiology Laboratory, Nebraska Medicine, Omaha, NE, USA

## Abstract

Whole genome sequencing (WGS) and clinical review were used to characterize 14 cases of central line-associated bloodstream infection (CLABSI) due to *Staphylococcus epidermidis*. WGS, which demonstrated disparate strains, suggested that 42.9% of *S. epidermidis* CLABSI cases were due to contamination, while clinical review suggested that 57.1% were contamination events.

## Introduction

Central line-associated bloodstream infections (CLABSIs) are linked with morbidity, mortality, and increased healthcare costs.^1^ CLABSIs are publicly reported and are often used as a metric for hospital safety, quality, and reputation.^2^ Owing to its ecological niche as a commensal organism of human skin, *Staphylococcus epidermidis* is a prominent cause of infection of vascular catheters and blood culture contamination. To reconcile these issues, the Center for Disease Control and Prevention (CDC) definition of CLABSI due to commensal skin microbes requires recovery of these organisms from at least 2 blood cultures, the presence of a central venous catheter (CVC), and lack of evidence of an explanatory infection at an alternative site.^3^ Catheter-related BSI (CRBSI) is a clinical definition that requires additional corroborating evidence such as local signs of infection, cultures of vascular catheter tips revealing the same organism as blood cultures, or a shorter time to positive culture (> 2 h earlier) from blood obtained via the incriminated vascular catheter compared to peripheral blood (differential time-to-positivity, DTP).

A CRBSI is presumed due to a single strain of bacteria, and thus organisms recovered from the peripheral blood, the CVC, and the catheter tip should all be identical.

Whole genome sequencing (WGS) can be used to determine the genetic identity of an organism and allows precise definition of genetic relatedness of one strain of bacteria to another,^4^ helping to differentiate between true cases of CRBSI and blood culture contamination.

Although the overall CLABSI rate decreased at our hospital from 2018 to 2023, the proportion of CLABSI caused by *S. epidermidis* increased from 27% to 55%. This shift may be attributable to effective CLABSI prevention efforts that decreased CLABSIs caused by other organisms, but we hypothesized that at least some of the CLABSIs were caused by repeated blood culture contamination. We utilized WGS to help characterize *S. epidermidis* CLABSI and to compare the genetic relationship with a clinical assessment of whether they were likely CRBSI.

## Methods

Setting: 680 bed academic medical center

*Staphylococcus epidermidis* CLABSI: 42 *S. epidermidis* blood culture isolates from the peripheral blood or CVC from 14 patient’s meeting the CDC-National Healthcare Safety Network (NHSN) criteria for CLABSI during 2023 were prospectively identified and saved for future genetic analysis.

Clinical review of CLABSI: two experienced infectious diseases physicians (MER, KAC) further reviewed *S. epidermidis* CLABSI cases to assess for likelihood of culture contamination or CRBSI. Clinical signs and symptoms, DTP, and factors potentially related to blood culture contamination (eg difficult phlebotomy, obesity/body mass index, underlying exfoliative dermatitis, etc.) were assessed. Cases were independently graded as likely CRBSI, likely contamination, ambiguous—more likely CRBSI, and ambiguous—more likely contamination. Discrepancies were further discussed, and consensus was established.

WGS and Multi-locus sequence typing (MLST): WGS was performed via Illumina iSeq100 using the Clear Labs sample prep and sequencing platform. MLST typing to determine sequence types (STs) of each isolate was done using the online tools provided by the Center for Genomic Epidemiology (https://cge.food.dtu.dk/services/MLST/). Genome assembly, phylogenetic analysis, and determination of genomic distances were done as previously described.^5^ For WGS determination of patients isolates as disparate stains (contamination) or same strain (CRBSI), we used a fifty single-nucleotide variations cutoff, which had been used in prior studies.^6^

## Results

**Figure 1.**
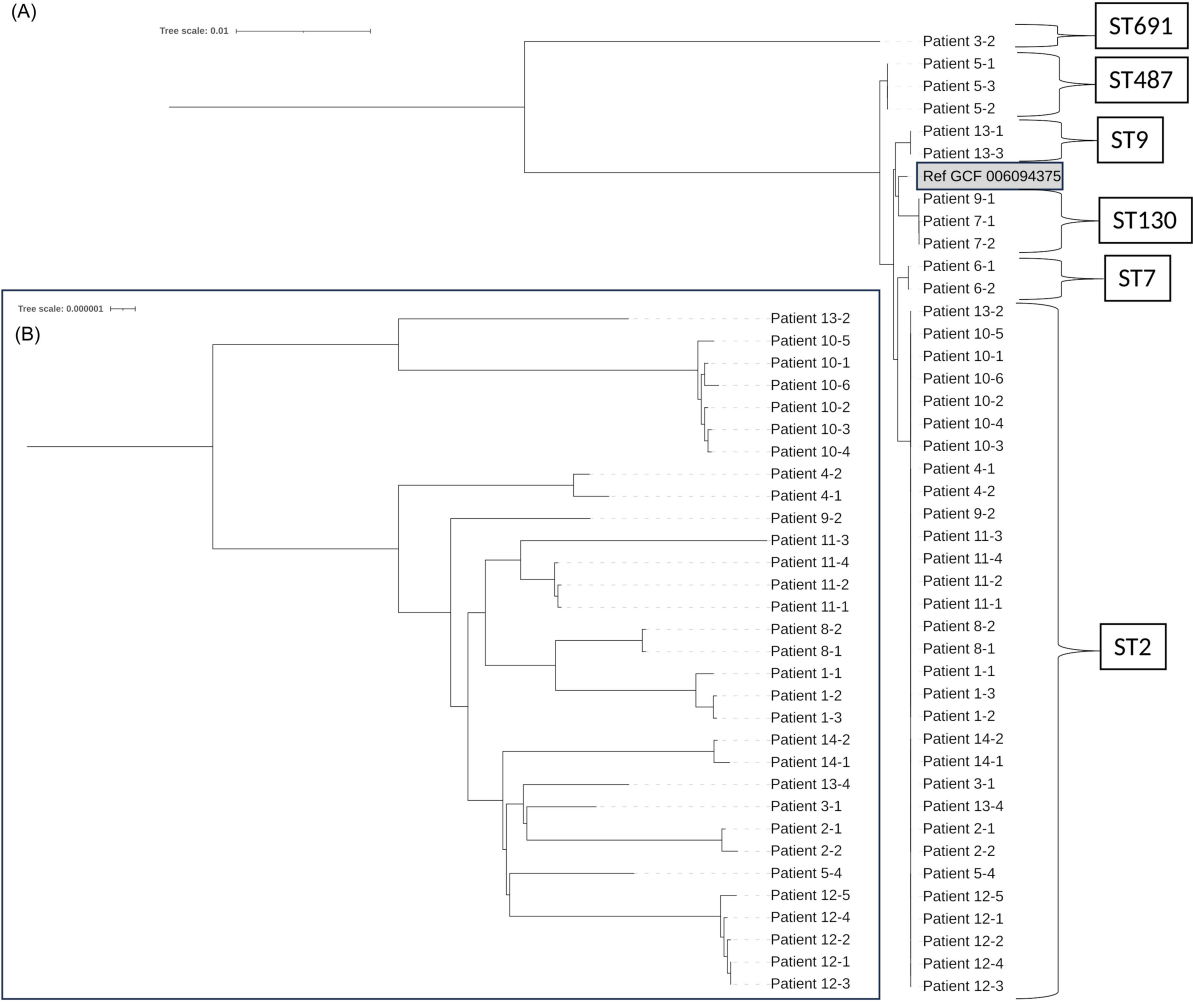
Phylogenic analysis revealed sequence types and genetic relatedness of the isolates. (A) Phylogenic tree containing all 42 isolates in the study. (B) Phylogenic tree of all 31 ST-2 isolates. (“Patient X-Y” indicates the “Y” ^th^ isolate from patient “X”; *S. epidermidis* GCF 006097375 was used as the reference strain).

*S.epidermidis* isolates were obtained only from the peripheral blood in 10 patients, the CVC and peripheral blood in 3 patients, and only the CVC in 1 patient. A summary of WGS results and clinical review is presented in Table 1 and Figure 1. Based on MLST analysis, sequence type 2 (ST2) was the most common among all isolates (N = 31, 70%), followed by ST130 (N = 3, 7%), ST487 (N = 3, 7%), ST7 (N = 2, 5%), ST9 (N = 2, 5%), and ST691 (N = 1, 2%). Genomic distances determined from phylogenetic analysis, favored contamination in 6 patients and favored CRBSI in 8 patients. When clinical review results were compared to WGS determinations, CRBSI determinations agreed in 2 of 2 cases (100%); Contamination determinations agreed in 2 of 4 cases (50%); Ambiguous, less likely CRBSI agreed in 3 of 4 cases (75%); Ambiguous, more likely CRBSI was in agreement in 2 of 3 cases (66%), and Ambiguous, unresolved was determined to be favoring CRBSI by WGS. Overall, clinical review and WGS agreed in 9 of 13 cases (69.2%). Of the 4 cases when clinical review and WGS disagreed, in 3 instances clinical review suggested contamination while WGS determination favored CRBSI; in 1 instance clinical review favored CRBSI, while WGS indicated contamination.

**Table 1.** Comparison of clinical review of *S. epidermidis* CLABSI and whole genome sequencing results

Patient Identifier	Number of Isolates	Clinical Review Result and CRBSI criteria	WGS Result	WGS Determination	Agreement between Clinical Review and WGS
1	3	Contamination	Same strain	CRBSI	No
2	2	Ambiguous, more likely CRBSI	Same strain	CRBSI	Yes
3	2	Ambiguous, less likely CRBSI	Disparate strains	Contamination	Yes
4	2	Ambiguous, more likely CRBSI	Same strain	CRBSI	Yes
5	4	Ambiguous, more likely CRBSI	Disparate strains	Contamination	No
6	2	Contamination	Disparate strains	Contamination	Yes
7	2	Contamination	Same strain	CRBSI	No
8	2	Contamination	Disparate strains	Contamination	Yes
9	2	Ambiguous, less likely CRBSI	Disparate strains	Contamination	Yes
10	6	CRBSI (CVC (+) x 3 days with (+) peripheral blood culture	Same strain	CRBSI	Yes
11	4	Ambiguous, unresolved	Same strain	CRBSI	N/A
12	5	CRBSI ((+) DTP)	Same strain	CRBSI	Yes
13	4	Ambiguous, less likely CRBSI	Disparate strains	Contamination	Yes
14	2	Ambiguous, less likely CRBSI	Same strain	CRBSI	No

WGS, whole genome sequencing; CRBSI, catheter-related bloodstream infection; DTP, differential time-to-positivity > 2 hours.

## Discussion

As the CLABSI definition is widely used for healthcare-associated infection surveillance,^7^ there is intense interest in improving the precision of the definition. Contamination of blood cultures obtained from CVCs is common and can be difficult to discern from CRBSI. Based on expert clinical review, 57% of CLABSI cases were judged ambiguous and 62.5% favored contamination. Based on WGS, 6 of 14 (43%) were determined to be due to contamination.

WGS of microbes has been extensively applied to clinical diagnostics, research, and epidemiological surveillance.^8,9^ This study showed that WGS was useful for the identification of STs of *S. epidermidis* and defined ST2 as being the most common sequence type in our isolate population. The ST2 sequence type has previously been noted to be responsible for ongoing nosocomial transmission.^6^ WGS was useful in more precisely defining genetic relatedness and demonstrating intra-patient and inter-patient strain variability. In some instances, identical strains were found from different patients (potentially indicating nosocomial acquisition) while in others, different strains were found in cultures from the same patient (more likely indicating blood culture contamination).

Disparities between the surveillance CLABSI definition, strain relatedness as determined by WGS, and review by clinical experts were noted in this study. Prior studies indicated considerable strain variation in *S. epidermidis* that made up the human skin microbiome.^10^ Therefore, this study suggested that WGS determination of different strains of *S. epidermidis* derived from multiple peripheral blood cultures or CVC/peripheral cultures was indicative of culture contamination and excluded CRBSI. Similarly, finding genetically identical strains of *S. epidermidis* in paired blood cultures favored the diagnosis of CRBSI. In 3 of 14 (21%) cases of CLABSI (patients 1, 7, and 14), clinical review favored the diagnosis of blood culture contamination or less likely CRBSI, which was excluded by WGS. In 1 instance (7%, patient 5), clinical review favored CRBSI diagnosis which was contradicted by finding disparate strains on WGS.

Our study has limitations. First, the criteria chosen to determine strain relatedness in the comparative genomic analysis can be debated. Fifty single-nucleotide variations were used as the cutoff for identical strains, which had been used in prior studies.^6^ However, this may not be the best measure for defining relatedness among *S. epidermidis* strains causing CRBSI. Second, being a single-centered study, with a relatively small number of cases and isolates, our results may not be generalizable.

*S. epidermidis* is a common cause of blood culture contamination and CRBSI, and differentiation between the two on clinical grounds is difficult. This study demonstrated that WGS can be applied to the definition of *S. epidermidis* CLABSI to better discern true CRBSI from contamination. Further investigation into the utility of WGS in *S. epidermidis* CLABSI is warranted.
